# A Comprehensive Study on the Influence of Sodium Chloride on the Technological Quality Parameters of Soft Wheat Dough

**DOI:** 10.3390/foods9070952

**Published:** 2020-07-17

**Authors:** Marina Carcea, Valentina Narducci, Valeria Turfani, Francesco Mellara

**Affiliations:** Research Centre for Food and Nutrition, Council for Agricultural Research and Economics (CREA), Via Ardeatina 546, 00178 Rome, Italy; valentina.narducci@crea.gov.it (V.N.); valeria.turfani@crea.gov.it (V.T.); francesco.mellara@crea.gov.it (F.M.)

**Keywords:** soft wheat, sodium chloride, dough, Brabender Farinograph, Chopin Alveograph, Rapid Visco Analyzer, Rheofermentometer, gluten

## Abstract

This study aimed at understanding how the presence or absence of NaCl influences dough rheological performance of soft wheat cvs. currently used in the Italian bread manufacturing industry as a scientific support to national health strategies to reduce the use of NaCl in bread. For this reason 176 flour samples belonging to 41 soft wheat cvs. currently cultivated in Italy, were analyzed for their protein content, Zeleny sedimentation value, and by means of the Chopin Alveograph and Brabender Farinograph, with no salt and with 1.5% salt addition (average salt content in Italian bread). Three selected cvs. (Aubusson, Bolero, and Blasco) were additionally studied by means of the Rapid Visco Analyzer (RVA) at three levels of salt addition (0%, 1.5% and 3.0%). The fermentation behaviour of the cvs. Aubusson and Blasco was also studied by means of a Rheofermentometer under the same conditions. The results of our study confirmed the role of salt in strengthening the wheat gluten network (up to 86%), and thus the gas retention of dough and in affecting yeast activity. However, it also definitely proved that careful cultivar selection can help in overcoming technical challenges in reduced-salt bread manufacturing and eventually, it opens the path to wheat breeding for reduced-salt bread baking.

## 1. Introduction

Sodium is essential for the human metabolism. However, current sodium intakes far exceed health recommendations [1]. Excess sodium intake is associated with hypertension, which is a major risk factor for cardiovascular diseases; it has been estimated that 62% of strokes and 49% of coronary heart diseases are caused by high blood pressure [2]. Moreover, high sodium intake has also been associated with several other negative health effects, including renal disease, renal stones [3], decreased bone mineral density [4], gastric cancer [5], asthma [6], and possibly obesity [7].

It was estimated that, in industrialized countries, 10–15% in average of sodium intake is naturally occurring in foods, 10–15% comes from discretionary use of salt (sodium chloride) during home cooking or at the table and the remaining 70–75% comes from processed foods where salt is used extensively for several purposes [8,9,10,11].

WHO recommends a maximum adult salt intake of 5 g/day [12]. Different strategies and policy options have been proposed to achieve this goal and several initiatives have been implemented especially in the European region, aiming at reaching this level of salt consumption [11,13,14,15].

Salt reduction in processed products has been proposed as a high-impact intervention for reducing the sodium intake at population level. Amongst processed foods, cereal products contribute the most to the overall salt intake, especially in those countries where they are staple foods [16,17,18,19].

In Italy, about 54% of salt intake is due to processed foods [20] and bread was identified as one of the targets of salt reduction initiatives within the European Salt Reduction Framework [21,22].

Salt has critical technological functions in dough and bread other than sensory contribution and its reduction can create significant processing challenges that the bread manufacturing industry has yet to face, for example, salt influences dough rheological behaviour and at reduced salt levels dough handling can be affected due to sticky dough phenomena, creating major processing issues and a poor quality final product [23]. Several salt substitutes have been experimented in bread to reduce sodium, but they often have negative effects on sensory properties [24].

In this context, a project was launched in Italy (“Strategies for improving competitiveness of Italian wheat production by reducing salt use in bakeries”, acronym EUSAL) with the purpose of acquiring scientific knowledge on a number of technological aspects of bread making that could help find effective and sustainable strategies for the reduction of salt use by the wheat processing industry besides educating consumers’ taste to bread with a reduced salt content. The project aimed at studying the variation in the technological behaviour of soft wheat doughs with varying salt (sodium chloride) content and try and relate this to cultivar characteristics, in order to evaluate the susceptibility of cultivars to be processed with a reduced quantity of salt and possibly gain a better understanding of the interactions between flour proteins and sodium chloride.

This study represents the first comprehensive report of technological behavior of wheat doughs belonging to many different genetic backgrounds in the presence or absence of salt and it could be used in guiding food industry and breeders in the selection of suitable genotypes for reduced-salt bread and bakery products manufacturing.

## 2. Materials and Methods

### 2.1. Grains and Flours

A number of 176 soft wheat (*Triticum aestivum* L.) grain samples belonging to 41 cultivars of different breadmaking quality (A416, Adelaide, Adelante, Africa, Afrodite, Albachiara, Altamira, Anapo, Anforeta, Apoteosi, Aquilante, Asuncion, Aubusson, Bandera, Bilancia, Blasco, Bolero, Centauro, Genesi, Guadalupe, Isengrain, Kalango, Masaccio, Mieti, Nogal, Nomade, Palesio, Pandas, PR22R58, Profeta, Quality, Sagittario, San Giacomo, Serpico, SO 207, Soissons, Stendal, Tiepolo, Trofeo, Vaiolet, and Zanzibar) and representing the Italian genetic soft wheat panorama currently used in bakery products, were collected at the farm over the harvest years 2011–2013 in Italy (65% coming from the central regions of Abruzzo, Emilia Romagna, Lazio, Marche, Toscana, and Umbria, and 30% from the northern regions of Friuli Venezia Giulia, Lombardia, Piemonte, and Veneto, and 5% coming from Sicily). For each cultivar, a minimum of 3 up to a maximum of 6 samples were collected, in different geographical locations whenever possible. Seeds were untreated. All samples were planted in late autumn, and rain-fed and harvested in early summer. Crop rotation and nutrient management (nitrogen and phosphorus, pre-sowing, and top-dressing fertilization) were managed by the farmers according to their usual practice.

The cultivar identity of all the samples was checked according to the analytical protocol described in the “Criteria for the inscription in the National Register of varieties of winter cereals (except rice)” [25].

Grain samples were tempered to 15.5–17.5% moisture (for 36–48 h depending on their hardness measured by means of the SKCS 4100 instrument, Perten Instruments, Stockholm, Sweden) and subsequently milled in a Bühler MLU 202 pilot mill (Canton St. Gallen, Bühler, Uzwil, Switzerland) equipped with three break rolls, three reduction rolls, and six screens, according to method 26–10.02 of the AACCI [26] to obtain commercial type white flours with an ash content of 0.55–0.65% (flours of 0 type according to the Italian official classification).

### 2.2. Chemical and Technological Analyses

Grain samples were analyzed for moisture by means of the Aquasearch P600 instrument (Kett Electric Laboratory, Tokyo, Japan). Flours were analyzed for moisture according to the ICC Method N.110⁄1 [27], for total protein by the Kjeldahl method according to AOAC Official Method 2001.11 [28] using 5.70 as conversion factor and for Zeleny sedimentation index according to the ICC Method 116/1 [27]. The Zeleny sedimentation value is supposed to range from about 8 for a very low protein, weak gluten type, to about 78 for a very high protein, strong gluten type. Ash content in flours was checked in a muffle furnace according to ICC Method N. 105/2 [27].

The effect of salt addition on the rheological properties of dough produced from the flour samples was investigated by means of the Chopin Alveograph (Chopin Technologies, Paris, France) and the Brabender Farinograph, equipped with a Sigma mixer S50 (50 g of flour) (Brabender, Duisburg, Germany), as follows.

The Chopin Alveograph test was run a first time according to the standard ICC Method n. 121 [27], then it was repeated by modifying the procedure in this way: pure distilled water was used instead of the sodium chloride solution prescribed by the method (1.5% salt in the dough with respect to flour weight). The two alveograms so obtained were compared with each other and the differences between parameters without and with salt were recorded (for a parameter *p*, Δ*p* = *p* with salt–*p* without salt). Relative differences with respect to the value without salt were also calculated and were expressed in percentage (per cent rΔ*p* = Δ*p*/*p* without salt ×100). In particular, the following parameters were measured: W (related to dough strength, i.e., the baking performance of a flour), P (related to tenacity, i.e., the resistance of the dough to deformation), and L (indicator of dough extensibility).

The Brabender Farinograph test was run a first time according to the standard ICC Method n. 115/1 [27] without salt addition, then it was repeated by modifying the procedure in this way: a sodium chloride solution was used instead of pure distilled water, to obtain a dough containing 1.5% salt with respect to flour weight. The two farinograms so obtained were compared with each other and the differences between Farinograph parameters with and without salt were recorded (for a parameter *p*, Δ*p* = *p* with salt–*p* without salt). Relative differences with respect to the value with no salt were also calculated and were expressed in percentage (per cent rΔ*p* = Δ*p*/*p* without salt ×100). The measured parameters were: water absorbance 14% m.b. (defined as the amount of water added to balance the Farinograph curve on the 500 Brabender Units (BU) viscosity line expressed as a percentage of the flour at 14% m.b.); development time (the time for the dough to reach its maximum viscosity); stability (indicating the strength of a flour, i.e., how well a flour resists overmixing,); and softening (indicates how fast the gluten structure breaks down after reaching its full development).

Flours obtained from grains of the cvs. Aubusson, Bolero, and Blasco, which are widely used in breadmaking in Italy and considered respectively as possessing weak, intermediate, and strong gluten, were also tested for their pasting properties by means of a RVA (Rapid Visco Analyzer) (Perten Instruments, Stockholm, Sweden) according to ICC Method n. 162, Standard 1 profile [27]. For each flour, the test was repeated according to a modified Standard 1 procedure in this way: 1.5% or 3.0% salt was added with respect to flour weight (the original procedure is without salt). The following parameters were measured: peak time, peak viscosity, through viscosity, viscosity breakdown, set back, and final viscosity.

Flours obtained from grains of the cvs. Aubusson and Blasco were furtherly tested for their fermentation profile by means of a Chopin Rheofermentometer (Chopin Technologies, Paris, France). The recommended Chopin protocol with slight modifications was followed: the Farinograph water absorption (ICC Method No. 115/1) [27] was used for optimum hydration and, respectively for each flour sample, both Rheofermentometer and Farinograph tests were repeated by adding 0%, 1.5%, and 3.0% salt with respect to flour weight. 

### 2.3. Data Presentation and Statistics

**Figure 1:**
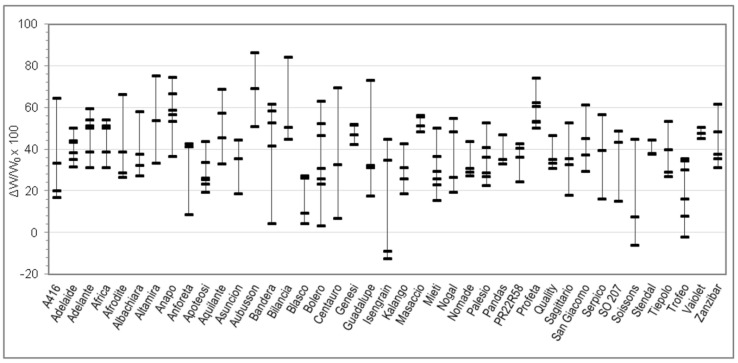
Percent relative difference between the Alveograph W registered by using salted water and the W registered by using distilled water (ΔW/W_0_ × 100 = (W*_without salt_* − W*_without salt_*)/W*_without salt_* × 100), for 176 samples of 41 soft wheat cvs (each dash on the graph represents the ΔW/W_0_ × 100 of a sample. Vertical lines join samples of the same cultivar).

**Figure 2:**
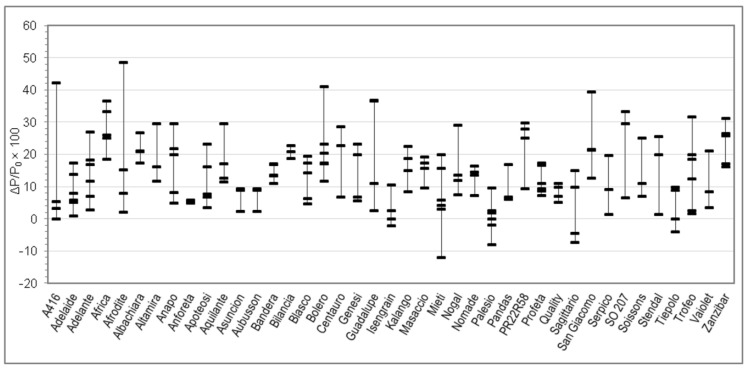
Percent relative difference between the Alveograph P registered by using salted water and the P registered by using distilled water (ΔP/P_0_ × 100 = (P*_with salt_* − P*_without salt_*)/P*_without salt_* × 100), for 176 samples of 41 soft wheat cvs (each dash on the graph represents the ΔP/P_0_ × 100 of a sample. Vertical lines join samples of the same cultivar).

**Figure 3:**
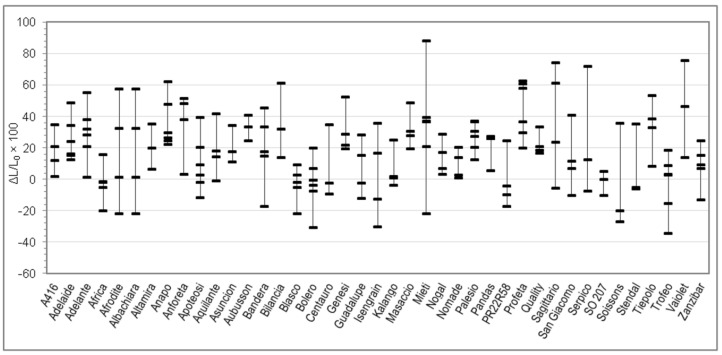
Percent relative difference between the Alveograph L registered by using salted water and the L registered by using distilled water (ΔL/L_0_ × 100 = (L*_with salt_* − L*_without salt_*)/L*_without salt_* × 100), for 176 samples of 41 soft wheat cvs (each dash on the graph represents the ΔL/L_0_ × 100 of a sample. Vertical lines join samples of the same cultivar).

**Figure 4:**
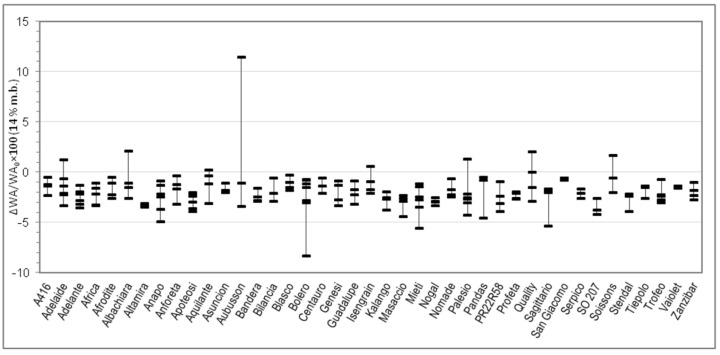
Percent relative difference between the Farinograph water absorbance (WA) 14% m.b. registered by using salted water and the WA 14% m.b. registered by using distilled water (ΔWA/WA_0_ × 100 = (WA*_with salt_* − WA*_without salt_*)/WA*_without salt_* × 100), for 176 samples of 41 soft wheat cvs (each dash on the graph represents the ΔWA/WA_0_ × 100 of a sample. Vertical lines join samples of the same cultivar).

**Figure 5:**
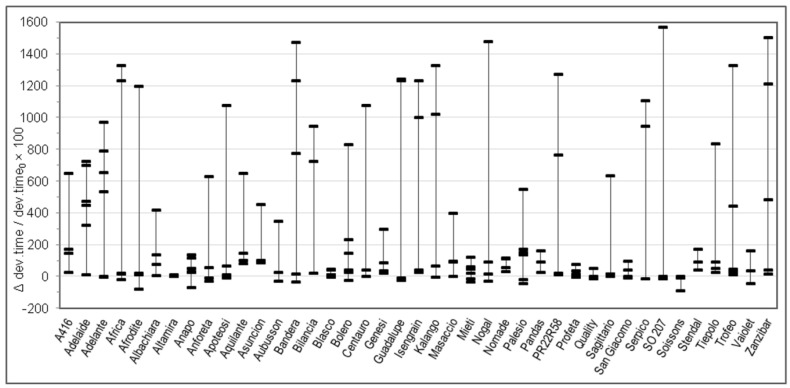
Percent relative difference between the Farinograph development time registered by using salted water and the dev.time registered by using distilled water (Δdev.time/dev.time_0_ × 100 = (dev.time*_with salt_* − dev.time*_without salt_*)/dev.time*_without salt_* × 100), for 176 samples of 41 soft wheat cvs (each point on the graph represents the Δdev.time/dev.time_0_ × 100 of a sample. Vertical lines join samples of the same cultivar).

**Figure 6:**
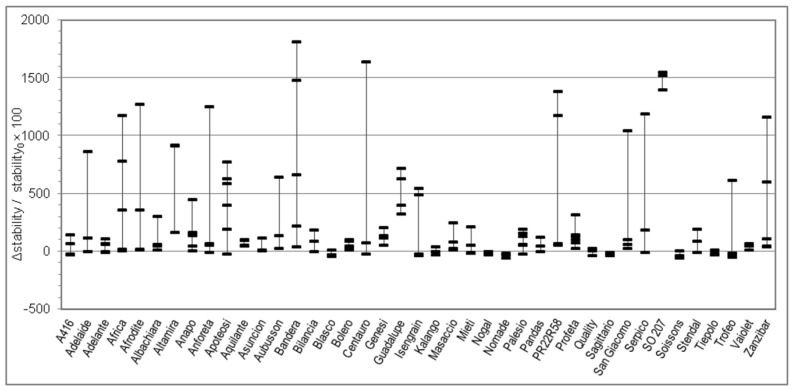
Percent relative difference between the Farinograph stability registered by using salted water and the stability registered by using distilled water (Δstability/stability_0_ × 100 = (stability*_with salt_* − stability*_without salt_*)/stability*_without salt_* × 100), for 176 samples of 41 soft wheat cvs (each point on the graph represents the Δstability/stability_0_ × 100 of a sample. Vertical lines join samples of the same cultivar).

**Figure 7:**
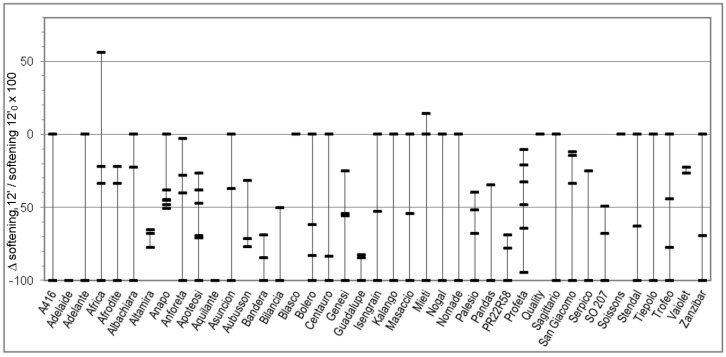
Percent relative difference between the Farinograph softening (12 minutes after maximum) registered by using salted water and the softening registered by using distilled water (Δsoftening12′/softening12′_0_ × 100 = (*_salt_* softening12′*_with salt_* − softening12′*_without salt_*)/softening12′*_without salt_* × 100), for 176 samples of 41 soft wheat cvs (each point on the graph represents the Δsoftening12′/softening12′_0_ × 100 of a sample. Vertical lines join samples of the same cultivar).

**Figure 8:**
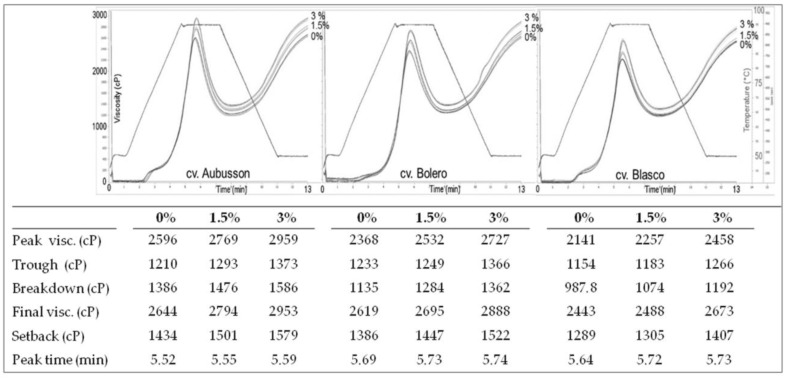
Rapid Visco Analyser (RVA) curves of flours of different bread making quality without salt and with 1.5% and 3.0% salt addition.

**Figure 9:**
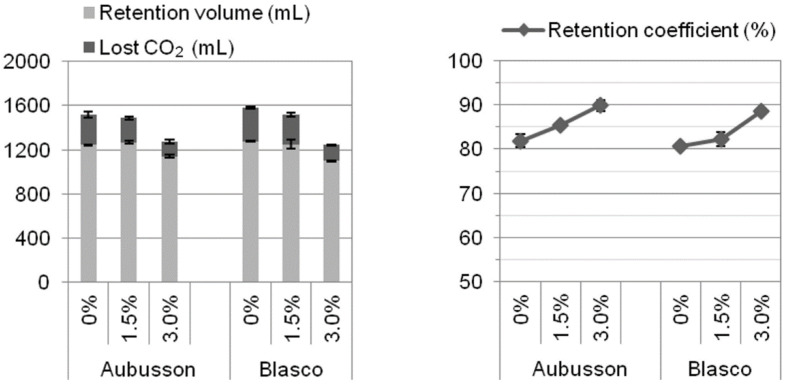
Retention volume, lost CO_2_, total volume *, and retention coefficient during fermentation without salt and at 1.5% and 3.0% salt addition for cvs. Aubusson and Blasco, respectively. * total volume = retention volume + lost CO_2._

On each sample, moisture, total protein, and Zeleny sedimentation index were analyzed in duplicate. Farinograph tests were performed in triplicate. Alveograph tests were performed in quintuplicate by default. RVA tests were performed at least in quadruplicate and Rheofermentometer tests at least in duplicate. Means of replicated determinations were than calculated for each sample. Table 1 reports min and max of the 176 samples, together with mean, SD, and percentiles calculated between the 176 samples. The per cent relative variation of each parameter upon salt addition (with respect to the value without salt) was then calculated for each sample and these numbers are represented in Figure 1, Figure 2, Figure 3, Figure 4, Figure 5, Figure 6 and Figure 7. Finally, data in Figure 8 and Figure 9 are means of replicated determinations for single samples of cvs. Aubusson, Bolero, and Blasco. Microsoft Excel and PAST statistical package version 2.17c [29] were used for calculations and statistics.

Since rheological variables were generally not normally distributed, non-parametric t-test (Wilcoxon Signed Rank Test) and non-parametric two-way analysis of variance were preferred; however, parametric tests (paired t-test and two-way ANOVA) gave the same result. T-test was applied on each single parameter, using salt level (0 or 1.5) as factor and the 176 samples as cases. Two-way analysis of variance was performed with salt level (0 or 1.5) and cultivar (all the 41 cvs. of the samples set) as factors and the 176 samples as cases.

## 3. Results

Quality parameters relative to the technological performance of all the soft wheat flour samples analyzed in this study are presented in Table 1 where descriptive statistics are reported for protein content, sedimentation value according to Zeleny, Alveograph parameters, and Farinograph parameters. Both the Alveograph and the Farinograph parameters are reported for salt-free dough and for dough containing 1.5% salt.

Summarizing the table data, we can say that protein content (% d.m.) ranged from 8.2 to 15.0 covering the range of values that are commonly found in wheat commercialized for bakery products manufacturing and, similarly, protein quality, as expressed by the Zeleny sedimentation Index value, went from 18 to 68 mL, lower values indicating poor bread baking ability; values below 20 are considered indicative of a flour unsuitable for bread baking.

Considering the Alveograph parameters obtained with salt the W went from 72 to 413 whereas values ranged from 42 to 342 in the absence of salt. A common classification agreed by the Italian industry and widely accepted by the bakers classifies the baking performance of flours according to the W index [30] as illustrated in Table 2.

The P and L values ranged respectively from 27 to 114 mm and from 40 to 233 mm in the standard procedure (with salt), and from 25 to 123 mm and from 27 to 183 mm in the salt-free procedure. Consequently, the P/L value (an indication of equilibrium between tenacity and extensibility) in standard conditions with salt was between 0.2 and 1.9, whereas in salt-free dough it went from 0.2 to 2.7.

Farinographic water absorbance (14% m.b.) is also reported in Table 1 for a dough without salt and with 1.5% salt; values ranged respectively from 48.6 to 62.8% and from 47.5 to 61.5%. The development time was also measured under the same conditions and it was between 1.0 and 20.0 min for the dough without salt and between 0.9 and 22.0 min for the dough with salt (Table 1). The stability was also measured in the Farinograph curve and it ranged between 0.9 and 21.4 min in the dough without salt and between 0.7 and 20.5 in the dough with salt (Table 1). The dough softening was also measured after 10 min and after 12 min (ICC Standard), without salt and with salt; after 10 min it went from 0 to 115 BU without salt and from 0 to 78 BU with salt. After 12 min it ranged from 0 to 131 BU without salt and from 0 to 82 BU with salt.

Considering the great variability observed in the quality parameters in Table 1 and in order to better understand the relationship between genotype and rheological behaviour of dough without and with salt, for each Alveograph and Farinograph parameter we reported also the percent relative difference registered by using salt-free and salted water for each analyzed sample in each cv. For each cv. we measured from three to six samples, coming from different cultivation areas. The W, P, and L parameters are studied in Figure 1, Figure 2 and Figure 3, respectively, whereas the water absorption (WA), development time, stability, and softening 12′ in Figure 4, Figure 5, Figure 6 and Figure 7, respectively. In these Figures, vertically aligned dash marks represent samples of the same cultivar. Dash marks below the zero line represent a decrease, whereas above the zero line represent an increase and next to the zero line mean substantially no variation (with respect to the parameter without salt). If the dash marks are close, it means that the relative variation of the parameter was similar for all samples of that cultivar. This was not the case for all cultivars because the analyzed parameters can also be influenced by sample growing conditions.

Figure 1 shows a very evident increase of the alveographic W, representing dough strength, in the dough with salt. Indeed, it was WNaCl > WøNaCl for 171 samples out of 176, representing 97% of samples (all points above zero except samples belonging to the cvs. Isengrain, Soissons, and Trofeo). The extent of the variation depends on the sample and the dispersion of values within samples of the same cv. is widely varying with the cv. We could identify the cv. Blasco as one with the smallest variation (no sample more than 27%) and Aubusson, Bilancia, Genesi, Masaccio, Profeta, and Vaiolet as those with the highest variation (no sample less than 40%), even if care should be taken in generalizing for cvs. because the number of samples for each cv. is limited.

Salt addition to the dough also produced an increase of the alveographic P, representing dough tenacity, in the great majority of samples (Figure 2). In fact, it was PNaCl > PøNaCl for 165 samples out of 176 (94%), whereas PNaCl = PøNaCl in 4 samples and PNaCl < PøNaCl in 7 samples. As for the W, the extent of the variation depended on the sample and the dispersion of values within samples of the same cv. varies with the cv.

For the parameter L, there were 19 cvs. which were L increased with salt in all samples, and 22 cvs. for which L increased only in part of the samples, whereas it decreased in the remaining part. In addition to the sign, the extent of the variation was still varying within the cultivar. The parameter G paralleled L as expected (data are not shown), since the two are strictly correlated.

Since the parameter P tended to increase with salt, whereas L increased in roughly three-quarters of cases and decreased in one-quarter and, moreover, the extent of the variation was not constant, one can understand that P/L varies without a defined tendency (data not shown); indeed, it increased with salt in 47% of samples and decreased in 51%. P/L was the only parameter for which the difference between the dataset P/LNaCl and the dataset P/LøNaCl was not statistically significant, which does not mean that it was not influenced by salt addition, but that the influence was more complex than for the other parameters, in fact it was indirect.

Considering the Farinograph parameters, salt addition produced a decrease in water absorption of a few percent points in 95% of samples (Figure 4). It also resulted in an increase—generally small, but high for certain samples—of development time (78% of samples, Figure 5) and stability (70% of samples, Figure 6). This was accompanied by a decrease in softening (72% of samples, Figure 7). The farinogram of strong flours like Blasco looked as if it was not affected that much by salt addition, whereas the curve of medium breadmaking quality flours as Aubusson was clearly improved and the curve of poor-quality flours was only slightly improved.

We then selected three widely used cvs. (Aubusson, Bolero, and Blasco, amongst all the cvs. studied) as representatives of different baking quality to study the behaviour of flours in the Rapid Visco Analyzer (RVA); in this case three levels of salt addition were experimented i.e., 0%, 1.5%, and 3.0%. The cvs. Aubusson and Bolero are classified in Italy as bread-making quality, and the cv. Blasco as superior bread-making quality, with Bolero being intermediate between Aubusson and Blasco. Total proteins of these flours were Aubusson 11.6% d.m.; Bolero 10.0% d.m.; and Blasco 9.9% d.m. The Alveograph W (with salt) was 367 for Blasco, 192 for Bolero, and 110 for Aubusson (whereas it was 336, 147, and 73, respectively, without salt). The RVA analysis was performed under standard conditions and the viscosity curves showing typical measurements for the cvs. Aubusson, Bolero, and Blasco are reported in Figure 8. At each salt concentration, viscosity at peak, through and end, together with breakdown and setback, were higher for Aubusson, intermediate for Bolero, and lower for Blasco. Progressive increase of salt concentration induced in all three cvs. a corresponding increase of viscosity at peak, through and end, and a slight delay of peak. It is interesting to notice that for all three cvs. the highest peak and final viscosities were reached for the maximum salt addition (i.e., 3.0%) whereas the 1.5% salt addition produced noticeable differences in the RVA curve with respect to no salt addition only for the Aubusson and Bolero cvs., whereas in the Blasco cv., the strongest quality one, the 0% salt curve and the 1.5% salt curves were almost superimposed.

**Figure 10:**
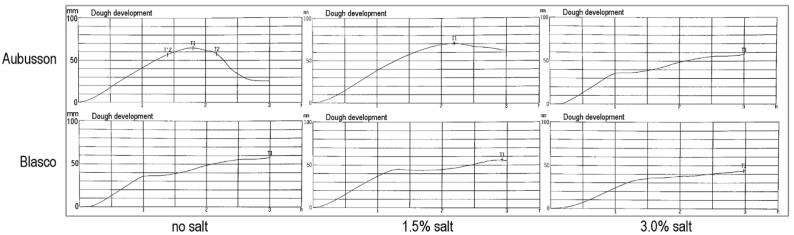
Dough development profile of cvs. Aubusson and Blasco during 3 h fermentation without salt and with 1.5% and 3.0% salt addition.

The two cvs. Aubusson and Blasco, which represented the extreme qualities in our pool of samples and which showed significantly different results in the rheology tests (RVA in particular), were then tested for their behavior during fermentation in the Rheofermentometer under the three salt levels, 0%, 1.5%, and 3.0%. Parameters related to the production of gas due to yeast action and CO_2_ release from the dough (total volume, retention volume, lost CO_2_, and retention coefficient) are reported in Figure 9, whereas the dough development profile during 3 h fermentation is reported in Figure 10.

Salt addition to the dough resulted in a decrease of gas production during fermentation and the dropping was much more pronounced at 3.0% salt concentration than at 1.5%. For the Aubusson flour, a dough made without salt produced a total gas volume of 1518 mL (Figure 9; total volume is the sum of retention volume with lost CO_2_). The addition of 1.5% NaCl led to a total volume of 1487 mL, whereas at 3.0% salt total volume dropped to 1272 mL. Similarly, for the Blasco flour, total volume was 1584 mL without salt, 1519 mL with 1.5% NaCl, and 1242 mL at 3.0% NaCl, respectively.

CO_2_ release from the dough also decreased with salt addition (Figure 9). Without salt, the Aubusson dough released 275 mL of CO_2_ and the Blasco dough 306 mL. At 1.5% salt, CO_2_ loss decreased to 218 mL for Aubusson and 270 mL for Blasco. At 3.0% salt, it dropped to 130 mL for Aubusson and 144 mL for Blasco. The retention coefficient, defined as (total volume-lost CO_2_)/total volume, increased with salt addition for both flours (Figure 9). For Aubusson, it passed from 81.9% at no salt, to 85.4% at 1.5% NaCl, and to 89.8% at 3.0% NaCl, respectively. For Blasco, values were 80.7% without salt, 82.2% with 1.5 NaCl. and 88.5% with 3.0% NaCl, respectively.

The dough development profiles during fermentation of the Aubusson and Blasco flours presented in Figure 10 show that without salt, the dough made from the weak Aubusson cv. rose to a maximum height first, then deflated sensibly. The dough made from the strong Blasco cv. rose continuously, with a pause (shoulder) but without deflating and reached a final height that was also its maximum height. The addition of 1.5% salt strengthened the Aubusson dough, that did not deflate sensibly anymore and its maximum height, which was like the one without salt, occurred later. On the contrary, there was no appreciable effect on the final height of the Blasco dough (the shoulder was slightly delayed and increased). At 3.0% salt addition, the profile of the Aubusson dough changed completely and became like that of the Blasco dough with no salt or 1.5% salt (rising continuously with a shoulder), whereas the Blasco dough became too tenacious and rose less than with no salt or 1.5% salt.

## 4. Discussion

The assessment of empirical dough rheological properties is a cornerstone of cereals evaluation worldwide and in Italy the Chopin Alveograph and the Brabender farinograph are widely used to estimate the breadmaking potential of flours which are classified according to the Farinograph and Alveograph parameters (see also the Results section) [30]. For this reason we chose to study with these two instruments a large number of flours coming from 41 different genotypes in the absence and in the presence of 1.5% salt as it might happen under normal processing conditions in an Italian bakery [22] to try and understand how the presence of such an amount of salt influences the flour behaviour compared to no salt (the ideal condition).

Table 1 shows some descriptive statistics of the main technological quality parameters of the analyzed samples. The Zeleny index spanning a wide range of values was a clear confirmation of the different breadmaking quality of our flours. The comparison of rheological parameters determined with varying salt content in the dough suggests that the presence or absence of salt does influence these parameters. In this regard, it is worth mentioning that the standard ICC Alveograph test is run on a dough containing approximately 1.5% salt; data are always reported for a dough with salt; therefore, our data for a dough without salt are totally new.

Indeed, t-test resulted in significant difference (*p* < 0.01) between all Farinograph and Alveograph parameters with or without salt, except for P/L, which is a calculated ratio (see Results section). Two-way analysis of variance confirmed this result and, as expected, showed that all parameters are influenced by the cultivar (*p* < 0.01), but did not confirm an interaction between the two factors (salt × cultivar). However, this is understandable since flour properties not only depend on genetic traits but are modulated by growing conditions of grains. Differences between parameters with and without salt resulted to be of quite variable extent between samples (Table 1), even belonging to the same cv. Because of this, and because of the small number of samples available for each cv. (3–6), it was found more convenient to investigate our results by plotting in the same figure the relative variations of parameters for single samples rather than compare means between cvs. (Figure 1, Figure 2, Figure 3, Figure 4, Figure 5, Figure 6 and Figure 7).

Changes of the Alveograph parameters (Figure 1, Figure 2 and Figure 3) show that salt addition generally strengthens the gluten network by increasing W, P, and (in two thirds of cases) L; thus, improving the viscoelastic characteristics of the dough except if the dough was already tenacious. The contemporary increase in farinographic development time and stability (Figure 4, Figure 5, Figure 6 and Figure 7) indicates that, in the presence of salt, the development of a stronger gluten network requires a longer development time and the network is fully developed at the end of the mixing process. This is related to the reduction of the hydration rate, which results into a longer duration of water absorption.

Several authors have investigated on the addition of salt to a wheat dough and how it affects its Farinograph parameters. Kojima et al. [31] reported a decrease in water absorption and an increase in the development time of the dough at a 1.5% salt level. When salt was added to the dough the protein association was increased, water absorption decreased but resistance to extension and extensibility increased. Ionic, hydrophobic, and hydrogen bonds are thought to be involved in these phenomena [32]. Beck at al. [33] also report that their Farinograph studies indicated that decreasing NaCl concentration increased water absorption and explained that this tendency confirms the assumption of decreasing protein hydration capacity due to the competition of sodium and chloride ions and water molecules on the protein surface. It is thought that Na as well Cl ions occupy the protein side chains overlaid at the beginning by bound water and therefore the flours water absorption decreases. The same authors also reported a decrease in dough stability by reducing NaCl content and explained this effect because of charge shielding by Na and Cl ions which does not allow the protein side chains to approach each other leading to a weaker gluten network formation and higher degree of softening.

When salt is added to a dough, Na^+^ and Cl^−^ ions can interact with polar binding sites of gluten protein and compete with water for them. This probably results in slowed gluten hydration and hence in increased development time, together with reduced water absorption. When salt was added as a solid to dough ingredients before mixing, the first part of the farinogram (dough development) was less smooth than when salt was previously dissolved in water, indicating that salt participates to the process of dough formation. Full-charged ions Na^+^ and Cl^−^ probably establish strong polar interactions with gluten proteins that lead to a stabilized gluten network, so that strength and machinability of dough is improved. It seems, however, that this effect can improve technological quality of flours of medium strength, but cannot change a too weak flour, whereas a strong flour would not need it.

It is undoubtful that the addition of NaCl produces rheological modification of dough primarily by influencing the formation and structure of the gluten matrix. The work by Beck et al. [33] investigated on the modifications to the gluten matrix microstructure of a single flour by NaCl by means of confocal laser-scanning microscopy (CLSM). Less linkages between proteins and elongated protein structures were found in the dough with 20 g NaCl kg^−1^ flour compared to 40 g NaCl kg^−1^ flour. When 0 NaCl was used, the protein structure changed from elongated protein strands to very less connected protein particles. These effects are due to the neutralization of charge repulsion emanating from charged amino acid moieties on the surface of gluten proteins. This charge neutralization is caused by sodium and chloride ions.

McCann and Day [34] studied the gluten network formation at the early stages of dough development by means of CLSM in doughs added with various amounts of NaCl produced by using two commercial wheat flour samples, a high protein flour, and a low protein flour. They concluded that the formation of gluten network is delayed in the presence of NaCl which was attributed to a reduction in the gluten hydration rate. This delay may impact on the protein unfolding, the alignment of protein polymers and subsequently the gluten structure. They observed that the presence of NaCl also produced the formation of elongated fibril protein structure at the end of dough development and that the effect of NaCl on the delay of dough development and other rheological parameters was different for the two flours; they suggested that the desired dough microstructure, optimum dough rheology and stability could be achieved with less amount of salt if the flour contains an appropriate quantity and quality of proteins.

Tuhumury et al. [35] studying two commercial wheat flours with different levels of total proteins in the presence or absence of NaCl by means of CLSM, transmission electron microscopy, and chemical analysis of disulfide bond linkages and the ratio of polymeric glutenins and monomeric gliadins, confirmed the fact that NaCl caused gluten to form fibrous structures and NaCl presence increased non-covalent interactions and β-sheet structure in gluten proteins. So, the presence of salt during dough mixing results in different molecular conformation and network structure of gluten proteins which contributed to the differences in the rheological properties.

Recently, Chen at al. [36] investigated on the disulfide, sulfhydryl groups, surface hydrophobicity, secondary structure, and extractable gliadin and glutenin of gluten in hard wheat flour doughs prepared with five different levels of NaCl. They found that the presence of salt decreased the free sulfhydryl content but increased the β-sheet structure of gluten. The extractable level of gliadin greatly decreased while glutenin increased with NaCl implying that more polymeric and less soluble protein networks were formed.

More recently a paper by Avramenko et al. [37] investigated on the role of NaCl level on the handling and water mobility in dough prepared from four different wheat cvs. and it was concluded that in terms of dough rheology, both cv. and NaCl levels were significant factors and their findings, in agreement with ours, indicate that careful cv. selection can help in mitigating challenges in dough handling within a reduced NaCl environment. Moreover, a paper by Fan et al. [38] studied the effect of NaCl on the rheological properties of dough and noodle quality obtained from three wheat cvs., possessing strong, intermediate, and weak gluten; they concluded that the addition of NaCl had different effects on the rheological properties of different varieties of wheat flour as we also observed.

The RVA is a rotational viscometer designed to test properties of viscous materials during cycles of heating/cooling under shear that simulate processing conditions. The viscosity changes of a suspension of flour in excess water under shear during a heating/cooling cycle between 50 °C and 95 °C, called pasting properties, are directly related to swelling of starch granules due to water absorption and to starch retrogradation. The protein fraction, however, modulates flour pasting properties by originating a gluten network that can be more or less strong and capable of absorbing water and holding starch granules. As Figure 8 shows, increasing salt concentration induced an increase in all viscosity parameters and a slight delay in peak viscosity. Such behavior is reported in the literature [39], and it must be due to the effect of salt on the protein fraction of the studied flour suspensions, since RVA viscosity parameters of isolated wheat starch are reported to be lowered, rather than increased, by salt addition [40].The Aubusson flour seemed to be more sensitive than Bolero and Blasco to salt addition at low salt concentrations, since for this flour the addition of 1.5% of salt resulted in a more pronounced viscosity increase than for the other flours.

The volume of bread depends on both the quantity of CO_2_ produced by the yeast and the dough’s gas retaining properties. Fermentation is due to yeast reproduction and to the ability of the dough to hold the gas liberated during the process and to dilate under gas pressure. Yeast reproduction is related to the presence in the dough of fermentable sugars and amylolytic enzymes that liberate maltose (a fermentable sugar), as well as to water activity. The gas holding capacity of the dough is related to dough rheological properties. The actual rising is related to gas production, gas holding capacity and dough elasticity.

Our results show that the gas production/gas holding curves of the dough samples indicate that salt addition to the dough induced a decrease of CO_2_ production and release and an increase of the retention coefficient (Figure 9). Both effects were more pronounced at higher salt concentration. These results suggest a slowdown of yeast activity and a decrease of dough porosity in the presence of salt. The same effects are reported also by Lynch et al. [17] who explained that due to the osmotic pressure and the action of sodium and chloride ions on the semi-permeable membrane of yeast cells, yeast growth is inhibited, and thus a reduction of salt levels causes an increased yeast activity and thus higher CO_2_ production. Beck et al. [41] also concluded that reducing NaCl produced higher yeast leavening ability and explained that at low concentrations NaCl provides a stimulatory effect on yeast leavening ability or a possible optimum between gluten strength and gas-holding capacity of wheat dough.

The rising of the strong Blasco dough is little affected by the addition of 1.5% salt (Figure 10) and is damaged, rather than helped, by the addition of 3.0% salt. The medium strength Aubusson dough, instead, is helped by the presence of salt to sustain a longer fermentation and this effect is visible at 1.5% salt already.

## 5. Conclusions

This is the first comprehensive study on the influence of salt addition on dough empirical rheological qualities predictive of bread baking quality in single variety flours; 176 samples from 41 different genotypes (cultivars) were studied possessing a wide range of qualities. It is undoubtful that salt influences dough behaviour and in general all flours improve their baking quality. The results of our study confirmed the role of salt in strengthening the gluten network and thus the gas retention of dough and in affecting yeast activity. Part of this dough strengthening effect is due to the decrease in the water absorption of the flour when NaCl is present. A small quantity of salt (1.5%) is enough to improve the technological quality of medium breadmaking quality flours, so that they can sustain longer fermentations and can be mixed for a longer time. Strong flours perform well without adding salt and too much salt (3.0%) can make them too tenacious. Quantities of salt above 1.5% are unnecessary to improve breadmaking quality of flours. 

This certainly opens to the reduction of salt use in breadmaking, in line with recommendations to improve public health by reducing salt intake in the diet. From our study it seems that it may be possible to partially compensate for the effects of reduced NaCl on dough rheology through selecting flour with suitable gluten quantity and quality. The observed differences on the rheological behavior of different flours in the presence of NaCl may be due to the extent of hydrogen bonding formation within the gluten network because of differences in protein content but mainly in the gliadins and glutenins composition. Certainly, our scientific data regarding salt functionality in a wide number of flours possessing different baking qualities can help bakers gain a deeper insight into effects on end-product, select the most suitable flour quality, and make technical choices for the processing of satisfactory sodium-reduced bakery goods. At the same time, these data can be used to guide soft wheat breeding programs to achieve the desirable flours for the formulation of salt-reduced bread and bakery products. A specific study on the characterization of protein subunits of glutenin and gliadins for a better knowledge of the genetic background of promising cvs. could be the next step.

## Figures and Tables

**Table 1:** Descriptive statistics of several technological quality parameters (protein content, Zeleny index, and Alveograph and Farinograph parameters with and without salt) characterizing 176 soft wheat flour samples belonging to 41 cvs.

	Min	Max	Mean	SD	25th Percentile	Median	75th Percentile
Protein (% d.m.)	8.2	15.0	11.4	1.3	10.4	11.3	12.1
Zeleny value (mL)	18	68	35	9	28	34	40
W _NaCl_ (10^−4^ J)	72	413	201	69	154	199	240
W _øNaCl_ (10^−4^ J)	42	342	147	57	105	142	184
P/L _NaCl_	0.2	1.9	0.7	0.3	0.4	0.6	0.8
P/L _øNaCl_	0.2	2.7	0.7	0.4	0.4	0.6	0.9
P _NaCl_ (mm)	27	114	65	19	48	64	79
P _øNaCl_ (mm)	25	123	58	18	43	56	69
L _NaCl_ (mm)	40	233	111	32	87	109	131
L _øNaCl_ (mm)	27	183	97	29	74	95	117
WA (14%mb) _øNaCl_ (%)	48.6	62.8	55.5	2.7	53.6	55.8	57.2
WA (14%mb) _NaCl_ (%)	47.5	61.5	54.4	2.8	52.7	54.2	56.2
Devel.time _øNaCl_ (min)	1.0	20.0	5.6	5.6	1.5	2.4	8.3
Devel.time _NaCl_ (min)	0.9	22.0	8.0	7.3	1.7	2.7	15.7
Stability _øNaCl_ (min)	0.9	21.4	9.8	6.3	3.9	9.1	15.6
Stability _NaCl_ (min)	0.7	20.5	14.6	4.8	11.2	16.3	18.8
Softening 10′_øNaC_ (BU)	0	115	31	24	12	27	44
Softening 10′_NaCl_ (BU)	0	78	17	14	6	15	24
Softening 12′_øNaCl_ (BU)	0	131	36	31	0	34	59
Softening 12′_NaCl_ (BU)	0	82	12	19	0	0	22

_NaCl_ dough containing 1.5% salt (standard for Alveograph parameters); _øNaCl_ salt-free dough (standard for Farinograph parameters); SD Standard deviation; WA water absorption; BU Brabender Units; d.m. dry matter.

**Table 2:** Classification of Italian soft wheat flours.

Flour Type	W	P/L	Protein	Stability
	(10^−4^ J)		(N × 5.7)	(Minutes)
Strong Flours	300	1 max	14.5	15′
Medium Flours	220	0.6 max	13.5	10′
Weak Flours	160	0.6 max	11.5	05′
Cake Flours	115 max	0.5 max	10.5 max	-
